# CCIP: predicting CTCF-mediated chromatin loops with transitivity

**DOI:** 10.1093/bioinformatics/btab534

**Published:** 2021-07-21

**Authors:** Weibing Wang, Lin Gao, Yusen Ye, Yong Gao

**Affiliations:** Department of Computer Science, School of Computer Science and Technology, Xidian University, Xi'an, Shaanxi 710071, China; Department of Computer Science, School of Computer Science and Technology, Xidian University, Xi'an, Shaanxi 710071, China; Department of Computer Science, School of Computer Science and Technology, Xidian University, Xi'an, Shaanxi 710071, China; Department of Computer Science, The University of British Columbia Okanagan, Kelowna, BC V1V 1V5, Canada

## Abstract

**Motivation:**

CTCF-mediated chromatin loops underlie the formation of topological associating domains and serve as the structural basis for transcriptional regulation. However, the formation mechanism of these loops remains unclear, and the genome-wide mapping of these loops is costly and difficult. Motivated by the recent studies on the formation mechanism of CTCF-mediated loops, we studied the possibility of making use of transitivity-related information of interacting CTCF anchors to predict CTCF loops computationally. In this context, transitivity arises when two CTCF anchors interact with the same third anchor by the loop extrusion mechanism and bring themselves close to each other spatially to form an indirect loop.

**Results:**

To determine whether transitivity is informative for predicting CTCF loops and to obtain an accurate and low-cost predicting method, we proposed a two-stage random-forest-based machine learning method, **C**TCF-mediated **C**hromatin **I**nteraction **P**rediction (CCIP), to predict CTCF-mediated chromatin loops. Our two-stage learning approach makes it possible for us to train a prediction model by taking advantage of transitivity-related information as well as functional genome data and genomic data. Experimental studies showed that our method predicts CTCF-mediated loops more accurately than other methods and that transitivity, when used as a properly defined attribute, is informative for predicting CTCF loops. Furthermore, we found that transitivity explains the formation of tandem CTCF loops and facilitates enhancer–promoter interactions. Our work contributes to the understanding of the formation mechanism and function of CTCF-mediated chromatin loops.

**Availability and implementation:**

The source code of CCIP can be accessed at: https://github.com/GaoLabXDU/CCIP.

**Supplementary information:**

Supplementary data are available at *Bioinformatics* online.

## 1 Introduction

The studies of Chromatin conformation capture technologies, such as Hi-C (Lieberman-Aiden *et al.*, 2009) and ChIA-PET (Fullwood *et al.*, 2009) unveil hierarchical chromatin higher-order structures at different genomic scales: chromosome territories (Lieberman-Aiden *et al.*, 2009), A/B compartments (Lieberman-Aiden *et al.*, 2009), topologically associating domains (TADs) (Dixon *et al.*, 2012) and chromatin loops (Li *et al.*, 2012; Rao *et al.*, 2014; Tang *et al.*, 2015). These structures are closely related to the transcriptional regulation of genes, the cell differentiation and development of organisms and the occurrence of diseases (Bonev and Cavalli, 2016; Rowley and Corces, 2018).

Recent studies show that the formation of TADs can be explained by the loop extrusion model, which reveals the relationship between CTCF mediated chromatin loops and TADs (Fudenberg *et al.*, 2016; Sanborn *et al.*, 2015). First, architectural protein CTCF is enriched at TAD boundaries (Dixon *et al.*, 2012). Second, two boundaries of a TAD are often associated with a CTCF loop (i.e. peaks at Hi-C contact matrix) whose pairs of CTCF motifs at the loop anchor regions are in the convergent orientation (i.e. convergent loops) (Rao *et al.*, 2014). Third, convergent loops are formed by the loop extrusion process and can explain the formation of TADs (Fudenberg *et al.*, 2016; Sanborn *et al.*, 2015). The loop extrusion model reveals the formation process of convergent CTCF loops: Cohesin, the structural maintenance of chromosomes (SMC) complex, extrudes the chromatin to form a loop until it is blocked by CTCF binding peaks whose motifs are in the convergent orientation (Banigan and Mirny, 2020; Fudenberg *et al.*, 2016; Sanborn *et al.*, 2015). Finally, CTCF-mediated chromatin loops can be split into chromatin contact domains (CCDs), which are TAD-like structures (Tang *et al.*, 2015). Besides convergent loops, there are many CTCF loops whose pairs of CTCF motifs are in the tandem orientation (tandem loops) (Tang *et al.*, 2015). Overall, convergent loops are found to be related to TAD formation and can be explained by the loop extrusion model. However, the formation mechanism and effects of tandem loops remain unclear (Zhang *et al.*, 2018).

Although the loop extrusion model can explain the formation of the individual convergent loop, it is unclear how multiple convergent loops connected to the same loop anchor are organized into complex higher-order structures (Banigan and Mirny, 2020). Recent studies have shown that two continuous CTCF loops exhibit transitivity, suggesting that the anchors of these loops are colocalized at a single spatial position (Rao *et al.*, 2014). Furthermore, multiple chromatin conformation capture technology (Quinodoz *et al.*, 2018) and super-resolution microscopy technology (Gu *et al.*, 2020) have demonstrated that CTCFs form clusters within the nucleus, which indicates that anchors of CTCF loops can locate at the same spatial position and form complex higher-order chromatin structures. Inspired by these studies, we conclude that, in addition to direct loops formed by loop extruding, there are also many loops (e.g. tandem loops) that are formed due to the transitive effect of the colocalization of their loop anchors.

Because it is difficult and costly to map CTCF-mediated chromatin loops, an accurate predicting method using existing functional genomic data, DNA sequence data and CTCF-mediated ChIA-PET data to predict CTCF loops in new cell types is an alternative way (Kai *et al.*, 2018). Recent studies have shown that CTCF-mediated chromatin loops can be predicted from one-dimensional functional genomic data and DNA sequence features (Kai *et al.*, 2018; Zhang *et al.*, 2018). However, they have failed to consider the higher-order structures of CTCF loops, and it is still unclear whether the transitivity can contribute to the prediction of CTCF loops.

We developed CCIP, a two-stage random-forest-based machine learning method, to predict convergent and tandem loops. Our two-stage approach makes it possible for us to integrate in the training process transitivity-related information, in the form of graph connecting probability (GCP), as well as standard functional genome data and genomic data. Experimental results showed that our method predicts CTCF-mediated chromatin loops more accurately than Lollipop (Kai *et al.*, 2018) and CTCF-MP (Zhang *et al.*, 2018) in three aspects (within individual cell types, across chromosomes and across cell types). Feature importance analysis demonstrated that transitivity-based features help predict CTCF-mediated chromatin loops. Furthermore, we proposed a transitive triple model to explain the formation of tandem loops. Overall, we have three main contributions: First, we found transitivity is a basic feature of CTCF-mediated chromatins loops and it is informative for predicting CTCF loops. Second, transitivity can explain the formation of tandem loops. Third, we proposed an accurate predicting method that helps explore CTCF-mediated chromatin structures in another cell type or cell state.

## 2 Materials and methods

### 2.1 Data collection and preprocessing

CTCF ChIA-PET raw data of GM12878, HeLa-S3, MCF-7 and K562 cell line was downloaded from NCBI GEO database (accession: GSE72816) and Encode portal (accession: ENCSR000CAC, ENCSR000CAD). We used the ChIA-PET2 (Li *et al.*, 2017) pipeline using the same parameters with Lollipop (Kai *et al.*, 2018) to process these row data and got initial CTCF mediated chromatin loops. CTCF and RAD21 ChIP-seq data for these cell types were collected from the NCBI GEO dataset and Encode portal (see Supplementary Table S2 for accession number). The position weight matrix (PWM) of the CTCF motif from humans was obtained from the JASPAR dataset (Fornes *et al.*, 2020). We used FIMO software (Bailey *et al.*, 2009) by default parameters to obtain all CTCF motif occurrences in the human reference genome (hg19). Branch-of-origin (Yokoyama *et al.*, 2014) of CTCF motifs were downloaded from the GitHub page of CTCF-MP (Zhang *et al.*, 2018) software (https://github.com/ma-compbio/CTCF-MP). We download SPRITE (Quinodoz *et al.*, 2018) data of the GM12878 cell line from the NCBI GEO database (accession: GSE114242). We download ChromHMM chromatin annotation data from UCSC (https://genome.ucsc.edu/cgi-bin/hgTrackUi?db=hg19&g=wgEncodeBroadHmm).

### 2.2 Sample generation

Both positive and negative samples for supervised learning were generated using the following steps. Step 1, we extracted ChIA-PET loops whose distance of anchors longer than 10 kb and shorter than 1 Mb because the distances of most loops are in this interval (Supplementary Fig. S8) (Kai *et al.*, 2018). Step 2, we extracted CTCF motifs which each bind one and only one CTCF ChIP-seq peak as valid loop anchors (Zhang *et al.*, 2018). Step 3, we extracted loops as positive samples whose loop anchors each bind one and only one valid loop anchor defined by step 1. In this way, we confirmed that each loop anchor of positive samples has one and only one CTCF and CTCF motif. Step 4, we generated candidate negative samples by randomly pairing valid loop anchors and filtering out loops that overlapped with any CTCF loops from ChIA-PET data. Step 5, we equally split convergent positive samples into ten bins according to their distance of motif pairs. Then, we split candidate negative samples of convergent loops into the same ten bins according to intervals determined by positive ones (Whalen *et al.*, 2016). Step 6, we randomly sampled final negative samples of convergent loops on the same sample size with positive ones in each bin (Zhang *et al.*, 2018). In this way, we get final negative samples of convergent loops with the same sample number and distance distribution compared with positive ones. We obtained final negative samples of tandem loops in the same way.

### 2.3 Feature extraction

In this step, we extracted features for both positive and negative CTCF loops. We first extracted features for loop anchors from the CTCF motif, CTCF ChIP-Seq and RAD21 ChIP-Seq data. FIMO software output the strand, score and matched sequence of each CTCF motif occurrences. The score measures the matching degree of the motif and the matched sequence. We encoded the plus strand as 1 and the minus strand as 0. We encoded the matched sequence to a binary vector array using One-Hot Encoding. Specifically, we encoded base A as [1, 0, 0, 0], T as [0, 1, 0, 0], G as [0, 0, 1, 0] and C as [0, 0, 0, 1]. Then, we concatenated the array for each base of the matched sequence and obtained the vector presentation of the matched motif sequence. For CTCF and RAD21 ChIP-Seq data, we extracted the signal values of CTCF and RAD21 that overlapped with the CTCF motif. The signal values measure the overall enrichment of the binding region. Besides, we obtained the age features of motif occurrence (branch-of-origin) from CTCF-MP, which has found that ancient CTCF motif occurrences are more likely to form CTCF loops. We extracted these features from the two anchors of each loop, respectively.

Then, we extracted in-between features by summarizing the features of anchors between loop anchors. In-between features include the sum of CTCF scores between loop anchors, the sum of CTCF signal values between loop anchors, the sum of plus-strand CTCF signal values between loop anchors, the sum of minus-strand CTCF signal values between loop anchors, number of CTCF motifs between loop anchors, number of plus-strand CTCF motifs, number of minus-strand CTCF motifs. Besides, the genomic distance between motif pairs was also included for predicting. Then we concatenated these features to a matrix to represent the samples. Along with the GCP features, we get ninety-seven features overall.

### 2.4 Definition and computation of GCP

According to the principle of network transitivity, if a loop anchor A connects to a loop anchor B, and B connects to a loop anchor C, anchor A is more likely to be connected to anchor C. This implies that the existence of a short alternative path between two anchors A and C increases the likelihood for them to have a direct interaction. We propose a measure GCP (the abbreviation of Graph Connecting Probability) to capture and quantify such influence of network transitivity on loop formation. We give the formal definition of GCP in the next paragraph.

Let G(V,E,p) be an edge-weighted CTCF-mediated chromatin network, where V is the set of anchors, E is the set of loops, and the edge weight function p:E→R associates each edge e with the predicated probability p(e) learned in the first stage of our learning method (See Section 2 section for more details). For a given edge e with two endpoints u and v, let Path(e) be the set of paths between u and v in the graph G−e obtained by removing the edge e from G. We define the graph connecting probability GCP(e) of edge e as

$$\mathrm{GCP}(e)=\underset{\mathrm{path}\;\in \;\mathrm{Path}\;(e)}{\max}\left(\prod_{i=0}^{k}p\left(e_{i}\right)\right)=\text{exp}\;\left(-\underset{\mathrm{path}\;\in \;\text{Path}\;(e)}{\min}\sum_{i=0}^{k}-\text{log}\;p\left(e_{i}\right)\right),$$

where $\mathrm{path}=(e_{1},e_{2},\ldots ,e_{k})$ is a sequence of edges. Because $0\leq -\text{log}(p(e_{i}))\leq +\infty$ we can regard this minimum problem as a weighted shortest path problem and solve it with Dijkstra’s Algorithm (Cormen, 2009).

### 2.5 Random Forest and feature importance score

Random forest (Breiman, 2001), an ensemble learning method for classification, uses multiple decision trees to predict and vote the labels of samples. The ensemble of various decision trees, which are trained from combinations of different sample subsets and feature subsets, enhances the accuracy and controls the over-fitting of the model (Tan *et al.*, 2006; Zhou, 2012).

We used the scikit-learn (Pedregosa *et al.*, 2011) implementation of random forest (https://scikit-learn.org/stable/modules/generated/sklearn.ensemble.RandomForestClassifier.html). Feature importance is an attribute of the trained ensemble model. Because we use Gini impurity (Tan *et al.*, 2006) as the split criterion of decision trees, the feature importance of a specific feature is computed as the total reduction of the Gini impurity brought by that feature.

### 2.6 Colocalization analysis of CTCF loop anchors

We assigned anchors of SPRITE (Quinodoz *et al.*, 2018) clusters to their nearest CTCF peaks and filtered out anchors with the genomic distance greater than 5k bp from its nearest CTCF peak. In this way, we got the CTCF clusters that interact with each other simultaneously. Then, we counted the number of CTCF clusters that contained each transitive triple and filtered out transitive triples whose count is less than 20. The remaining transitive triples were spatial co-localization validated by multiple chromatin interaction data. If a tandem loop was contained in a transitive triple, we classified it as a valid sample, otherwise a non-valid sample. Then, we tested if positive tandem loops tend to be valid samples by Fisher’s exact test.

## 3 Results

### 3.1 Transitivity of CTCF-mediated chromatin interaction network

**Fig. 1.**
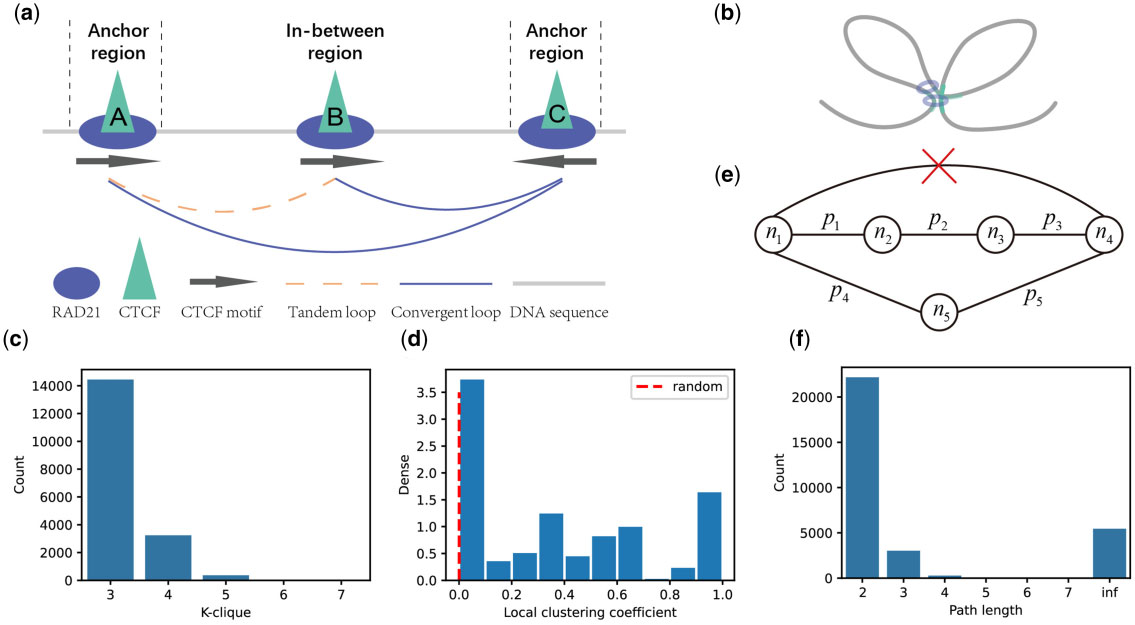
Transitivity of CTCF-mediated chromatin interaction network. (**a**) Illustration of transitivity. A, B, C denotes three loop anchors at specific DNA regions that occur CTCF motifs and bind CTCF proteins. CTCF motif pairs that are in tandem orientation are defined as tandem loops, such as loop (B, C) and loop (A, C). CTCF motif pairs that are in convergent orientation are defined as convergent loops, such as loop (A, B). For loop (A, C), we define anchor regions as CTCF binding regions and the in-between region as the region between these two anchor regions. (**b**) Illustration of transitivity using a 3D diagram, corresponding to (a). (**c**) K-clique number distribution of CTCF-mediated chromatin interaction network. (**d**) Local clustering coefficient distribution. The dashed red line represents the average local clustering coefficient of a random shuffled network which keeping the node’s degree unchanged. (**e**) Schematic illustration of alternative path length. (**f**) Alternative path length distribution

To determine whether the transitivity is a basic feature of CTCF-mediated chromatin loops, we analyzed the transitivity of the CTCF-mediated chromatin interaction network. We defined the CTCF binding regions as nodes and CTCF loops that connect these nodes as edges. In network science, network transitivity is a property that if node u connects to node v, and node v connects to node w, then node u connects to node w (Newman, 2018; Zafarani *et al.*, 2014). Similarly, in the CTCF-mediated chromatin interaction network, transitivity arises if two CTCF anchors, A and C, are co-located (and are more likely to form a loop) whenever CTCF A is spatially co-located with CTCF C (CTCF A and CTCF C form a CTCF loop), and CTCF B is co-located with CTCF C (Fig. 1a and b). In this way, such a transitive triple forms a spatial focus, which is the structural basis for transcriptional regulation (Tang *et al.*, 2015).

Perfect transitivity occurs only in a fully connected graph that rarely exists in real-world network data (Newman, 2018). However, partial transitivity is prevalent in the CTCF-mediated chromatin interaction network due to the existence of an abundance of k-cliques (Newman, 2018) (Fig. 1c). In graph theory, k-clique is a fully connected subgraph that has k vertices. Each clique may indicate the colocation of its nodes. For example, we found that there are 14 445 3-cliques and 3237 4-cliques (Fig. 1c). On the other hand, when we randomly shuffled the edges of the networks but kept the node degree unchanged, only several hundreds of 3-cliques were found in the resulting random network. Compared to nodes in a random network, we observed that many nodes in the chromatin interaction network have a large local clustering coefficient (Newman, 2018; Zafarani *et al.*, 2014), a standard transitivity measure used in network science to characterize the density of interactions between neighbors of a node (Fig. 1d).

Our observations, together with recent studies on the role of transitivity in the loop formation process, suggest the existence of alternative paths between a CTCF pair formed by the transitivity effect (Fig. 1ef). Specifically, if we remove the edge corresponding to the transitive loop, there should be other paths connecting the pair of anchors. An example of alternative paths is shown in Figure 1e. $N_{k}$ is the node of graph G, and $p_{k}$ is the probability of each edge linked to its two nodes. When we remove the direct path that links $n_{1}$ and $n_{4}$, we can also achieve $n_{4}$ from $n_{1}$ by $\mathrm{path}(n_{1},n_{2},n_{3},n_{4})$ and $\mathrm{path}(n_{1},n_{5},n_{4})$. We define the paths that can achieve $n_{4}$ from $n_{1}$ extruding $\mathrm{path}(n_{1},n_{4})$ as alternative paths of $\mathrm{loop}(n_{1},n_{4})$. We defined the length of the shortest path as the alternative path length. We computed the alternative path length for each loop and found that most loops have a short alternative path with the path length less than seven. There are also more than five thousand loops whose alternative path length is infinite (no alternative path), which indicates that these loops are formed in a direct way other than by the transitive effect. We think that the existence of a short alternative path is a necessary condition for the formation of transitive loops, and we can use this property to predict CTCF-mediated chromatin loops.

### 3.2 Overview of CCIP

**Fig. 2.**
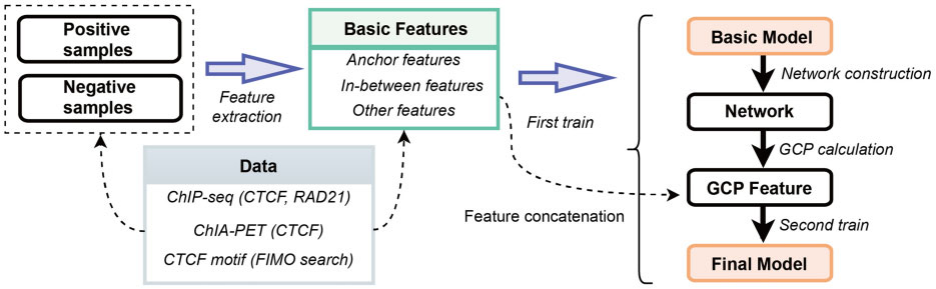
Overview of CCIP. The number of positive and negative samples is balanced

To determine whether transitivity is predictive for CTCF loops, we proposed a random-forest-based method (Breiman, 2001; Tan *et al.*, 2006; Zhou, 2012). The main steps are as follows (Fig. 2). First, we generated positive and negative samples of four cell lines (GM12878, HeLa-S3, K562, MCF-7) from CTCF ChIA-PET data, CTCF ChIP-seq data and CTCF motif occurrence data. Specifically, we used CTCF ChIP-seq data and CTCF motif occurrence data to define the candidate of CTCF loop anchors (Fig. 1a). Anchor pairs that are connected by CTCF ChIA-PET loops were defined as positive samples; other pairs were defined as negative samples.

Then, we extracted features for each loop using CTCF and RAD21 ChIP-seq data and CTCF motif occurrence data. CTCF and RAD21 are the main players in the loop extrusion model. Specifically, CTCF functions as the block element for loop extrusion, and RAD21, which is a component of Cohesin, functions as the molecular motor for loop extrusion (Banigan and Mirny, 2020; Fudenberg *et al.*, 2016; Sanborn *et al.*, 2015). Features were divided into three categories: anchor features, in-between features and other features. Anchor features extracted from loop anchor regions include CTCF motif strand, CTCF motif score, CTCF motif sequence (19 bp, One-Hot Encoding), CTCF motif age, CTCF signal value and RAD21 signal value. In-between features are extracted from the regions between the two loop anchors include the number of CTCF peaks, summary of CTCF signal, number of RAD21 peaks, etc. Other features include the genomic distance of loop anchors. We extracted in-between features motivated by recent studies which have found that CTCF binding peaks locate between loop anchors can repress the process of loop extrusion (Kai *et al.*, 2018) and features extracted from regions between loop anchors help predict enhancer–promoter interactions (Whalen *et al.*, 2016) and CTCF loops (Kai *et al.*, 2018).

Finally, the random forest model is trained by a two-stage strategy. In stage one, we trained a basic model by the features extracted above and the model predicts the probability of each sample being positive. The predicted probabilities of these samples are used to construct a CTCF-mediated chromatin interaction network whose weights of edges are these probabilities. From the network, we extracted the GCP features. GCP is the probabilistic version of the alternative path length (see Section 2 for more details). In stage two, the basic features extracted from the above steps and GCP features are combined to train a refined final model. Random forest classifier at these two stages is used because of its great generalization for heterogeneous features (Tan *et al.*, 2006). More details of the CCIP are discussed in Section 2.

### 3.3 Performance evaluation of CCIP

We compared CCIP with Lollipop (Kai *et al.*, 2018) and CTCF-MP (Zhang *et al.*, 2018). Lollipop predicts CTCF loops by training a random forest classifier, which extracts functional genomic features and sequence features. CTCF-MP predicts convergent CTCF loops by training a boosted tree classifier, which extracts DNA sequence features using the word2vec (Mikolov *et al.*, 2013) model. Lollipop uses all CTCF loops as training data while CTCF-MP only uses convergent CTCF loops. Besides, methods of generating positive and negative samples for Lollipop and CTCF-MP are also different. For fairness of comparison, we generated samples for these two methods by the same steps with CCIP (see Section 2 for more details).

**Fig. 3.**
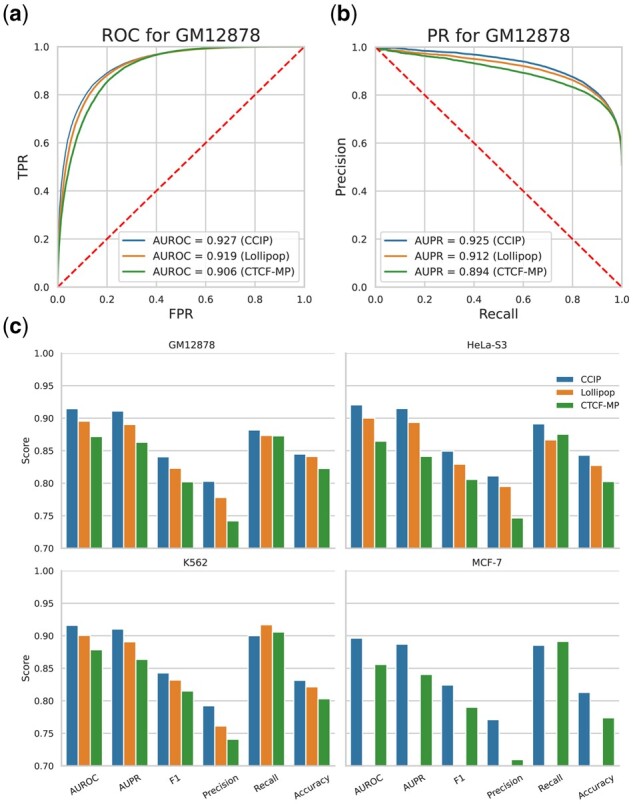
Performance evaluation of CCIP. (**a**) Receiver Operating Characteristic Curve (ROC) of GM12878 cell line. Within cell type evaluation. Ten-fold cross validation. (**b**) Precision–Recall Curve (PR) of GM12878 cell line. (**c**) Across chromosome evaluation using multiple performance metrics. At each cell line, we use some chromosomes as positive samples, and others as negative samples

We evaluated these methods within individual cell types by 10-fold cross-validation. Specifically, at each round of cross-validation, we randomly split the samples from a cell type to training datasets and testing datasets. The experimental results showed that CCIP achieved the best performance in GM12878 cell line compared with Lollipop and CTCF-MP (Fig. 3a and 3b), regardless of the area under the receiver operating characteristic curve (Tan *et al.*, 2006) (AUROC) or area under the precision–recall curve (Tan *et al.*, 2006) (AUPR). We did not compare Lollipop in the MCF-7 cell line, due to lack of functional genomic data in the cell. CCIP always achieved the best performance in these four cell types (Supplementary Fig. S1). We also evaluated these methods on the samples of convergent loop and tandem loop respectively and found CCIP has achieved better performance on both the convergent loops and the tandem loops (Supplementary Fig. S2).

We evaluated performance across chromosomes by 10-fold cross-validation. Specifically, we randomly chose some chromosomes as training chromosomes, and others as testing chromosomes at each round of cross-validation. We found that despite the difference in topological complexity of each chromosome due to the difference in gene density, the CTCF loops of each chromosome can be well predicted by this cross-validation strategy (Supplementary Fig. S3). Comparing with within individual cell type evaluation, all the performance metrics of CCIP decrease slightly (average 0.5%). However, CCIP still achieved the best performance in most cases (Fig. 3c).

Finally, we evaluated performance across cell types. Specifically, we used samples from one cell type as the training data, the other cell types as the testing data. In most cases, CCIP achieved the best AUROC and AUPR scores, except when the MCF-7 cell line was the training set and the K562 cell line was the testing set (Supplementary Figs S4 and S5).

### 3.4 Transitivity is predictive for CTCF loops

Machine learning methods can not only predict the labels of unknown samples but also help us determine features that are helpful for prediction (Tan *et al.*, 2006). In this section, we discuss the determinators of CTCF loops and discuss whether GCP is informative for predicting CTCF loops.

**Fig. 4.**
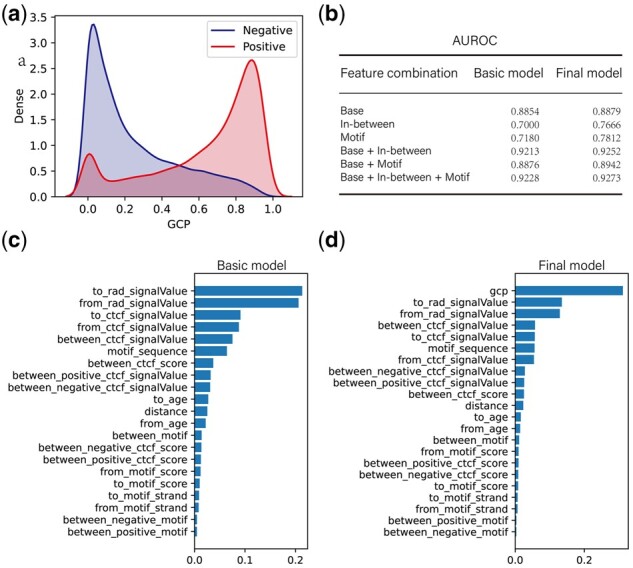
Feature importance analysis. (**a**) GCP distribution of positive and negative samples. (**b**) Feature combination and its performance (AUROC). Motif features are extracted from the CTCF motif sequence (One-Hot Encoding). Base features include anchor features but exclude motif features. (**c**) Ranks of feature importance for Basic Model. (**d**) Ranks of feature importance for Final Model

GCP features that were extracted from the CTCF-mediated chromatin interaction network can separate positive samples from negative ones (Fig. 4a). In most cases, positive samples have higher GCP scores than negative ones. However, some positive samples have a smaller GCP score, mainly due to these loops are derived from loop extrusion directly.

We combined the different types of features to determine whether each type of feature can improve the performance of the model (Fig. 4b, Supplementary Table S1). We repartitioned all the features into three categories: base, in-between and motif. Motif Features are extracted from the CTCF motif sequence (19 bp, one-hot encoding). Base Features include anchor features but exclude motif features. For all feature combinations, the final model that includes the GCP feature has higher performance than the basic models, especially for Motif and In-between feature combinations (6% improvement). This indicates GCP can improve the performance of the basic model.

Benefit from the advantage of random forest classifier, we can get feature importance scores from the trained model (see Section 2 for more details). Figure 4c and d shows the rankings of feature importance scores for the basic model and the final model, respectively. In the basic model, RAD21 and CTCF signal values at anchor regions are the most prominent features, consistent with the loop extrusion model (Fudenberg *et al.*, 2016) and Lollipop (Kai *et al.*, 2018). Next, the ranking of CTCF motif sequence features is extremely high which agrees with the results of CTCF-MP (Zhang *et al.*, 2018). However, it should be noted that we combine all importance scores from CTCF motif sequence features because 76 features total are extracted from the CTCF motif sequence (19 bp, one-hot encoding). If we rank all these features with other features directly, we will ignore the importance of the CTCF motif sequence. Besides, in-between features extracted from regions between loop anchor regions are also important. However, in the final model, the GCP feature score ranks first and it is greater than the sum of second and third features. This implies that GCP is a very predictive feature for predicting CTCF loops. Besides GCP, the ranking of other features is the same as the basic model. Overall, transitivity is informative for predicting CTCF loops.

### 3.5 The transitive triple can explain the formation of tandem loops

**Fig. 5.**
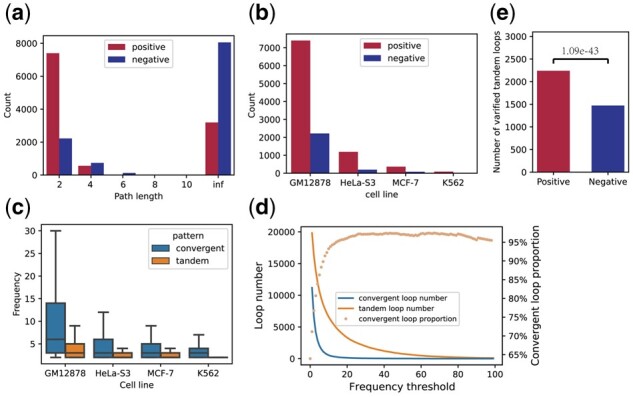
Transitive triples can explain the formation of tandem loops. (**a**) The alternative Path length of positive and negative tandem loops. (**b**) The number of tandem loops that are associated with transitive triples. (**c**) Interaction frequency of tandem and convergent loops. (**d**) The number and proportion of convergent loops and tandem loops at different frequency thresholds. (**e**) The number of positive and negative tandem loops that are associated with transitive triples and are spatial colocalization captured by multiple chromatin interaction data (*P*-value: 1.09e-43, Fisher’s exact test)

Increased evidence shows that convergent loops are generated by the loop extrusion model (Banigan and Mirny, 2020; Fudenberg *et al.*, 2016; Sanborn *et al.*, 2015). However, there is not a reasonable model to explain the formation of the tandem loops. We want to know whether transitivity can explain the formation of tandem loops. Through data analysis, we found the following five facts: First, positive tandem loops usually have shorter alternative path lengths than negative ones in the network constructed by convergent loops (Fig. 5a). This suggests that transitive tandem loops can be formed from the network of convergent loops. Second, the positive samples of the tandem loops are more likely to exist in the transitive triples than the negative ones (Fig. 5b). Third, the tandem loops usually have a lower interaction frequency than the convergent ones, which implies that the tandem loops are likely indirect interactions formed by transitivity (Fig. 5c). Fourth, as the threshold of interaction frequency increases (simulated decrease of sequencing depth), the number of tandem loops decreases faster than convergent loops (Fig. 5d). When the frequency threshold is 2, the convergent loop accounts for 65% of all loops, but when the threshold rises to 20, the convergent loop accounts for more than 95% (Fig. 5d). Lastly, transitive triples of positive tandem loops tend to spatially co-localize compared with negative tandem loops, which are observed by multiple chromatin interaction data (Quinodoz *et al.*, 2018) (Fig. 5e). Spatial colocalization of transitive triples is a prerequisite for transitive loops.

Recent studies found that 2–8 CTCFs are organized as a spatial focus, indicating that some loops can be formed by the transitive effect (Gu *et al.*, 2020). Transitivity can also explain the fact that tandem loops tend to locate within convergent loops observed by a recent study (Tang *et al.*, 2015). Overall, compared with the convergent loops, the tandem loops not only have a lower interaction frequency but also are more difficult to be captured with the decrease of sequencing depth. Moreover, colocalization of transitive triple and network of convergent loops form the basis for the transitive tandem loops. Therefore, tandem loops may be formed by the transitivity of convergent loops, and these loops are captured by the ligation step during the ChIA-PET sequencing process.

### 3.6 CTCF loops serve as the scaffold for enhancer–promoter interaction and transcriptional regulation

Recent studies have found that anchors of tandem loops are enriched with more active epigenomic markers, RNAPII and TSS densities than convergent loops (Tang *et al.*, 2015). This indicates that tandem loops are more likely involved in enhancer–promoter interactions. If tandem loops are the transitive effect of convergent loops, enhancer–promoter interactions associated with tandem loops may need a third CTCF as a mediator. Specifically, both enhancer and promoter interact with this third CTCF respectively by two convergent loops. In this way, both the enhancer and the promoter are pulled to the same spatial position and interact with each other.

First, we annotated all CTCF loop anchors with enhancers, promoters and CTCFs using ChromHMM (Ernst and Kellis, 2012) annotation data. We found 2032 CTCFs are annotated as promoters, 9355 CTCFs are enhancers. This indicates that CTCF is involved in gene regulation by binding at regulatory element regions.

Then, we detected 445 transitive triples that enhancer–promoter can be connected by a tandem loop while enhancer and promoter can be connected to the third element by convergent loops separately. We found that most of the transitive triples are in the TAD (79%), which indicates that the transitive triples are more subtle structures than the TADs (Supplementary Fig. S6a). TADs were detected by MSTD algorithm (Ye *et al.*, 2019) and have an average size of 500k.

**Fig. 6.**
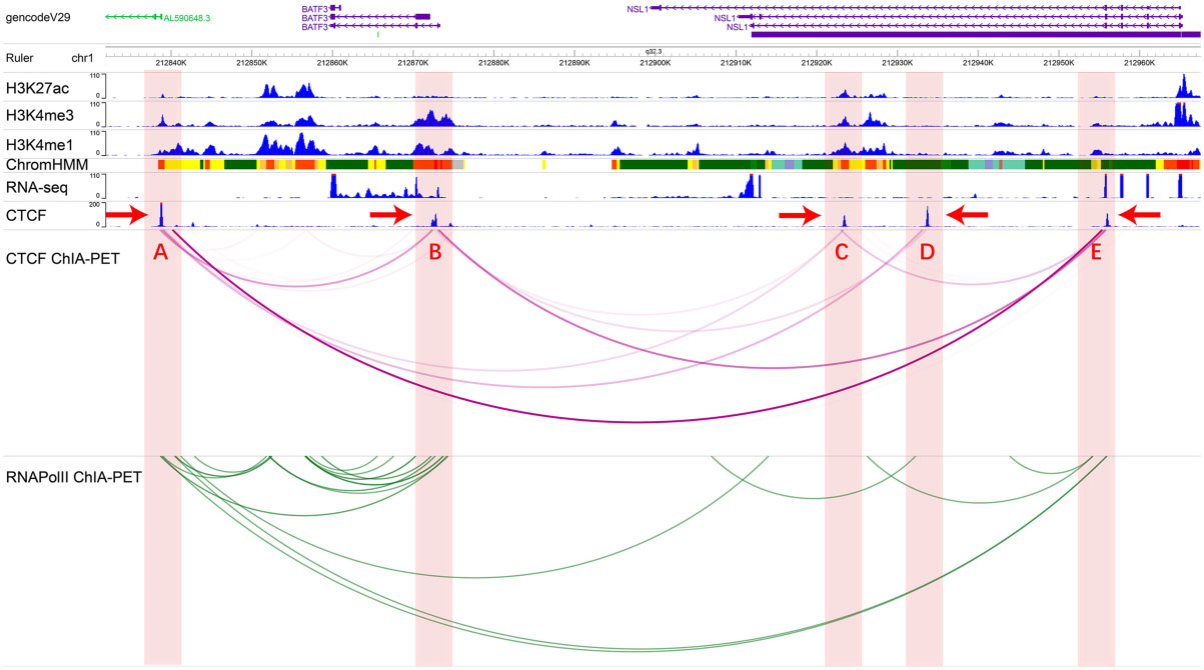
Genome browser snapshot of GM12878 near BATF3 gene. Red vertical bars highlight five CTCFs that are connected by CTCF loops and RNAPII loops. Red arrows indicate the orientation of CTCF motifs. BATF3 gene near the CTCF B. CTCF A and CTCF B are all connected to CTCF E. CTCF A, B and C constitute a transitive triple. CTCF A and CTCF B are annotated as the enhancer and the promoter respectively by the ChromHMM software. In the ChromHMM track, red represents promoter, yellow and orange represent enhancer and green represents transcription. This snapshot was generated by WashU Epigenome Browser (http://epigenomegateway.wustl.edu/browser/)

Figure 6 illustrates an example of tandem loops involved in gene regulation in the GM12878 cell line. Five CTCF peaks span over 140 kb genomic region within a TAD (Supplementary Fig. S6b). CTCF A, CTCF B and CTCF C are related to CTCF D and CTCF E by convergent loops. CTCF A and CTCF B are anchors of a tandem loop and they are annotated as enhancer and promoter, respectively. Through RNAPOLII ChIA-PET data, we can validate that the enhancer interacts with the promoter. CTCF E plays a key role that closes the spatial distance between the enhancer and the promoter and promotes their interaction. At the same genomic region of the HeLa-S3 cell line, the convergent loop that connects CTCF B with CTCF E, and the tandem loop that connects CTCF A with CTCF B is missing accompanying by the silence of the gene BATF3 (Supplementary Fig. S7). Together, CTCF loops play a key role in transcriptional regulation.

## 4 Discussion

Our observations on the highly transitive and clustered network structure of CTCF-mediated chromatin loops, together with the co-localization properties of CTCF anchors, motivated us to study the possibility of making use of transitivity-related information of interacting CTCF anchors to computationally predict CTCF loops. We designed a two-stage random-forest-based method for predicting convergent and tandem loops by using transitivity-based features. In stage one, our method predicts CTCF-mediated chromatin loops using network-free features extracting from one-dimensional functional genome data and genomic data in a way similar to the two recently proposed methods such as Lollipop and CTCF-MP. In the second stage, we compute the proposed GCP scores using the prediction from the trained model in the first stage and combine the GCP scores with other features to train a more accurate random-forest model. Although both CCIP and Lollipop use the random forest as the basis of the model, the main differences are as follows: (i) CCIP mainly focuses on the contribution of transitivity-based features to predicting the CTCF loop. Therefore, we designed a feature GCP that measures transitivity and proposed a two-stage prediction model. (ii) In addition to epigenetic features (CTCF and RAD21 binding), we also added one-hot-encoded sequence features to explore the influence of sequence features on the prediction results. (iii) CCIP only relies on CTCF and RAD21 ChIP-Seq data and DNA sequence data as the source of sample features, while Lollipop relies on more functional genomic data, which extends the application scenarios of our method. Despite relying on less data, CCIP still achieves better performance, especially when comparing across cell lines.

We analyzed the contributions of GCP for predicting. Our results showed that GCP is the most predictive feature in the second training stage, indicating that transitivity is important for the formation of some indirect loops. Besides GCP, we found that CTCF and RAD21 binding strength at anchor regions and between anchor regions are important for the model which is consistent with the loop extrusion model and recent studies. Besides, the CTCF motif conservation score and CTCF motif sequence are also informative for predicting which is consistent with recent works.

Our results and analyses indicate the more detailed formation process of CTCF-mediated chromatin loops: First, the DNA sequences where the CTCF motif occurs can specifically bind CTCF proteins, and the conservation of the motif occurrence determines the binding strength of CTCF. Second, RAD21 takes part in loop extruding, and thus direct loops are formed. Third, loop extrusion causes the co-localization of CTCF sites, forming CTCF foci. CTCF loci shorten the spatial distance of CTCFs including those CTCF sites that do not directly interact. Indirect loops are also captured by the ChIA-PET pipeline because of the short distance between CTCF loop anchors located at the same CTCF loci.

Our study provides much evidence to support the idea that tandem loops may be formed by the transitive effect. First, we found that positive tandem loops have shorter alternative path length on convergent loop network compared with negative ones which indicate that tandem loops can be formed by the transitive effect of convergent loops. Second, using higher-order chromatin interaction capture data, we found that positive transitive triples are enriched for SPRITE clusters compared with negative ones which indicates that CTCFs of transitive triples are co-localization indeed. Recent studies show that tandem loops tend to locate between convergent loops and tend to be associated with enhancer–promoter interaction. Because tandem loops are formed by the transitive effect of the third CTCF anchor, we believe that the enhancer and the promoter can get close to each other by the third CTCF anchor. In this way, enhancer and promoter are co-localized with CTCF loci and form a structure like a transcriptional factory, as has been suggested in a recent study. We leave it as future work to understand more details of these structures and their functions in transcriptional regulation.

## Supplementary Material

btab534_Supplementary_Data
