# Supplementary material for: CCIP: predicting CTCF-mediated chromatin loops with transitivity
Source: Bioinformatics. 2021 Jul 21;37(24):4635–42. doi: 10.1093/bioinformatics/btab534 (PMC8665748; doi:10.1093/bioinformatics/btab534)
Supplement: btab534_Supplementary_Data [file btab534_supplementary_data.docx]

Supplementary Materials

# Supplementary Figures


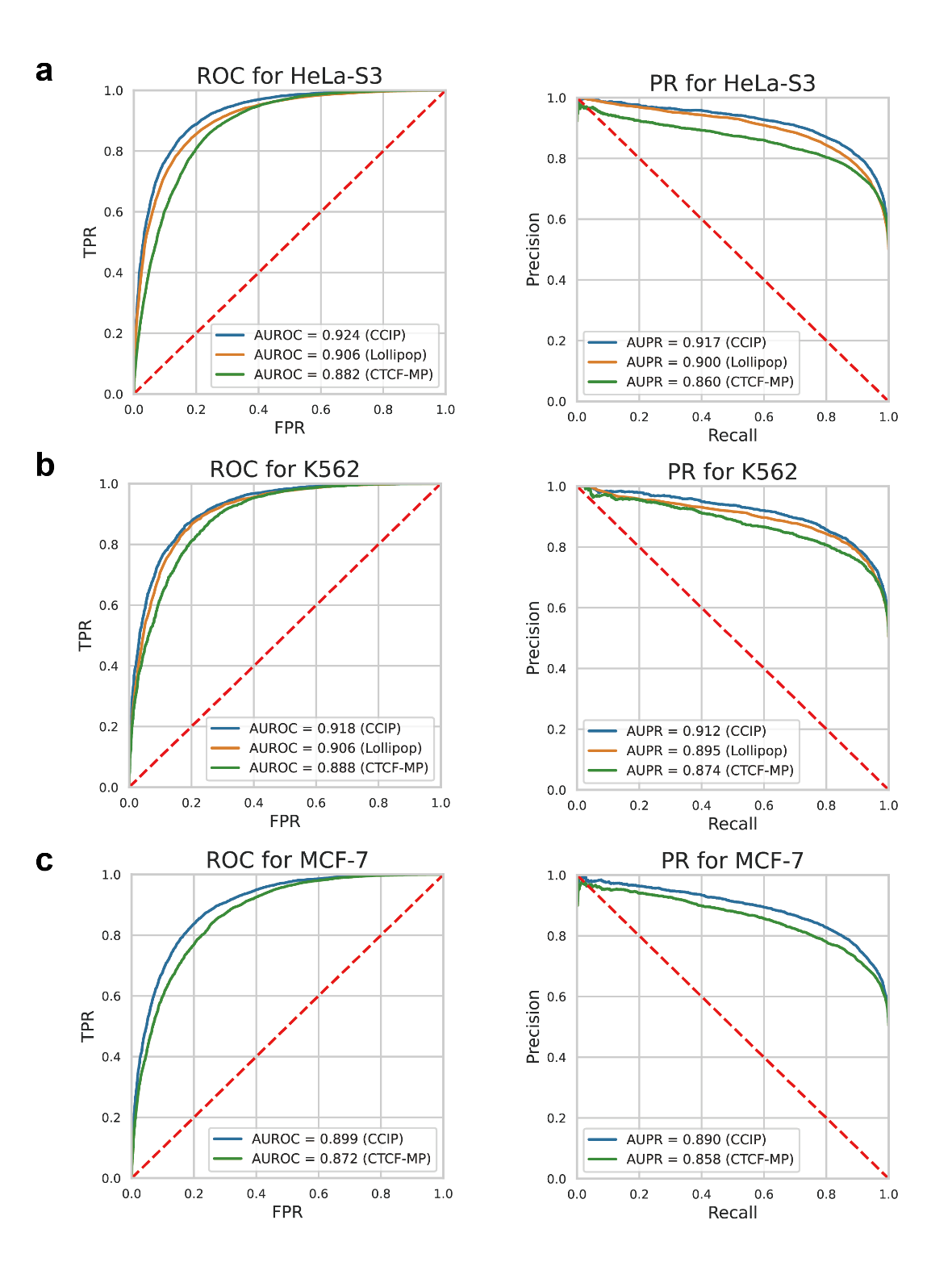


**Fig. S1. ROC curve and PR curve for HeLa-S3, K563, and MCF-7 cell line.** Within cell type evaluation. Ten-fold cross evaluation.


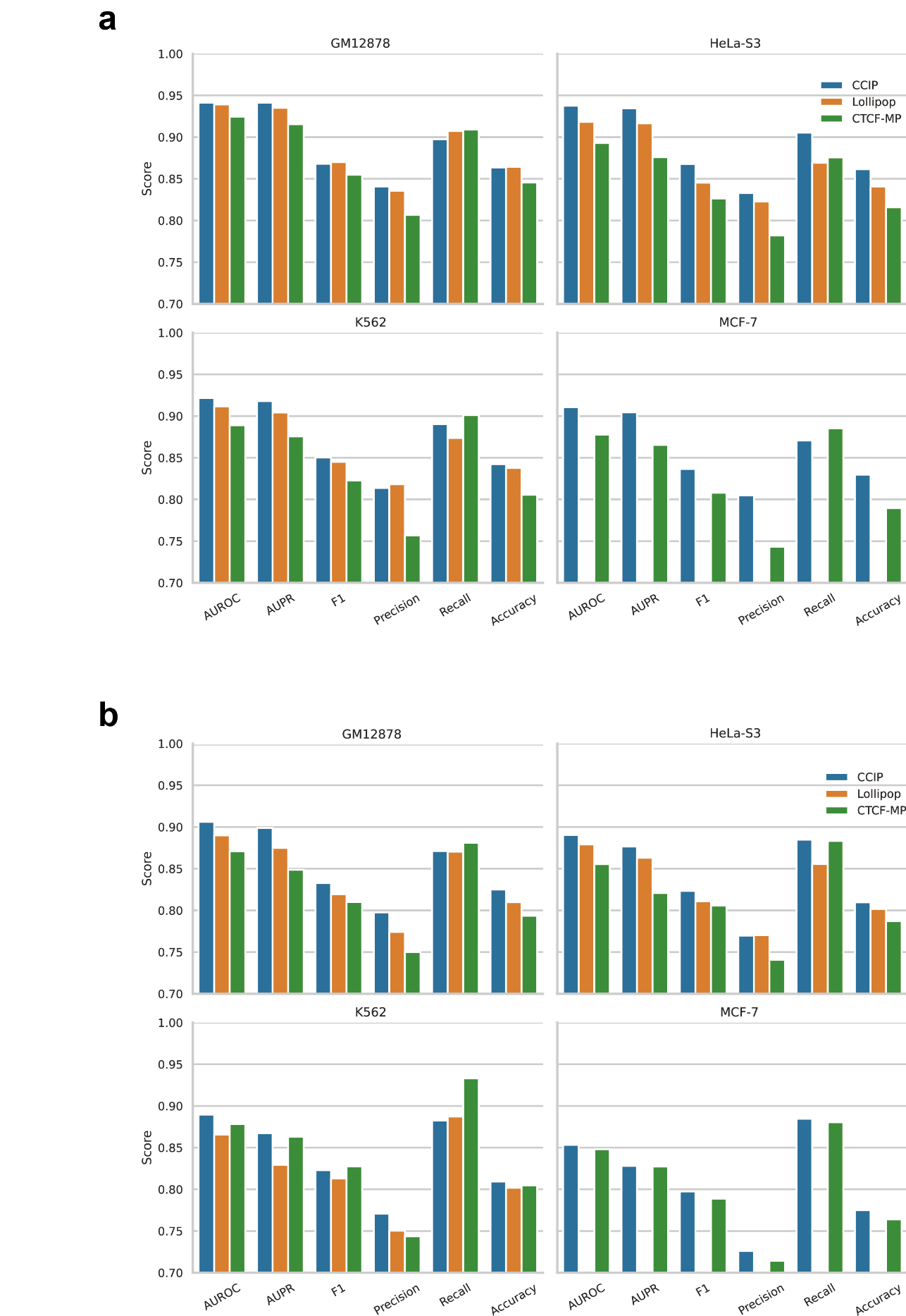


**Fig. S2. Evaluation on convergent loops and tandem loops.** (a) Evaluation on convergent loops. (b) Evaluation on tandem loops.


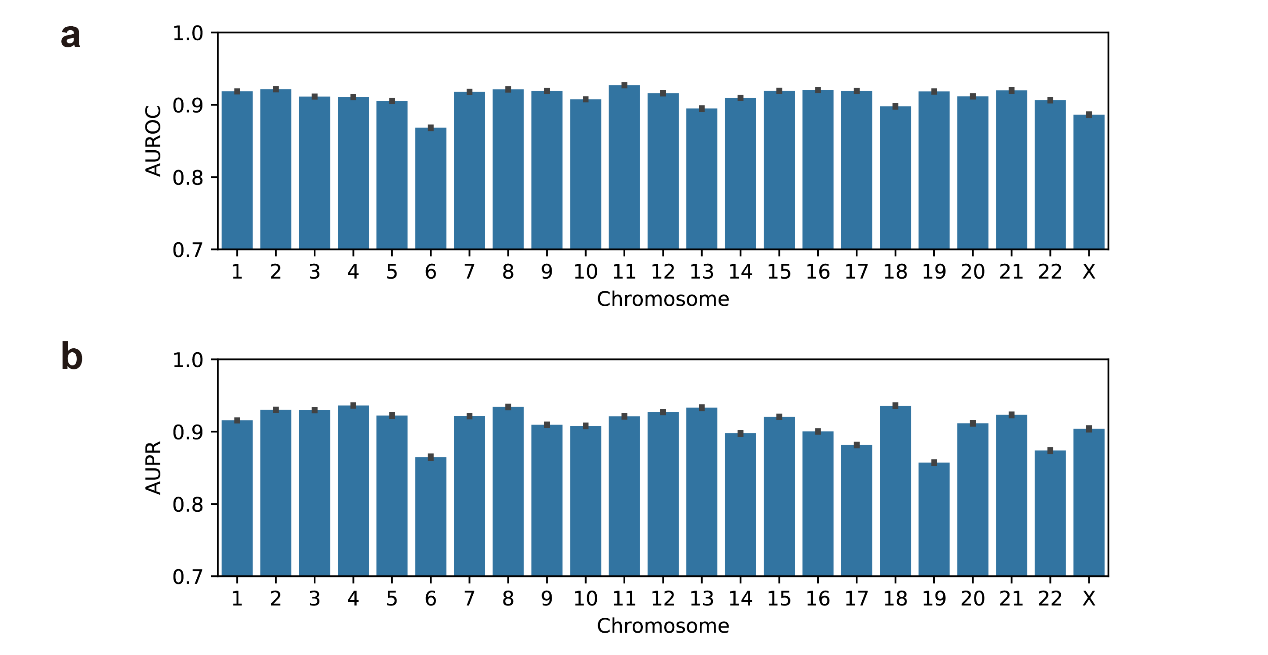


**Fig. S3. Performance for each chromosome.** We performed 10-fold cross validation 20 times with different randomization in each repetition. (a) AUROC score for each chromosome. (b) AUPR score for each chromosome.


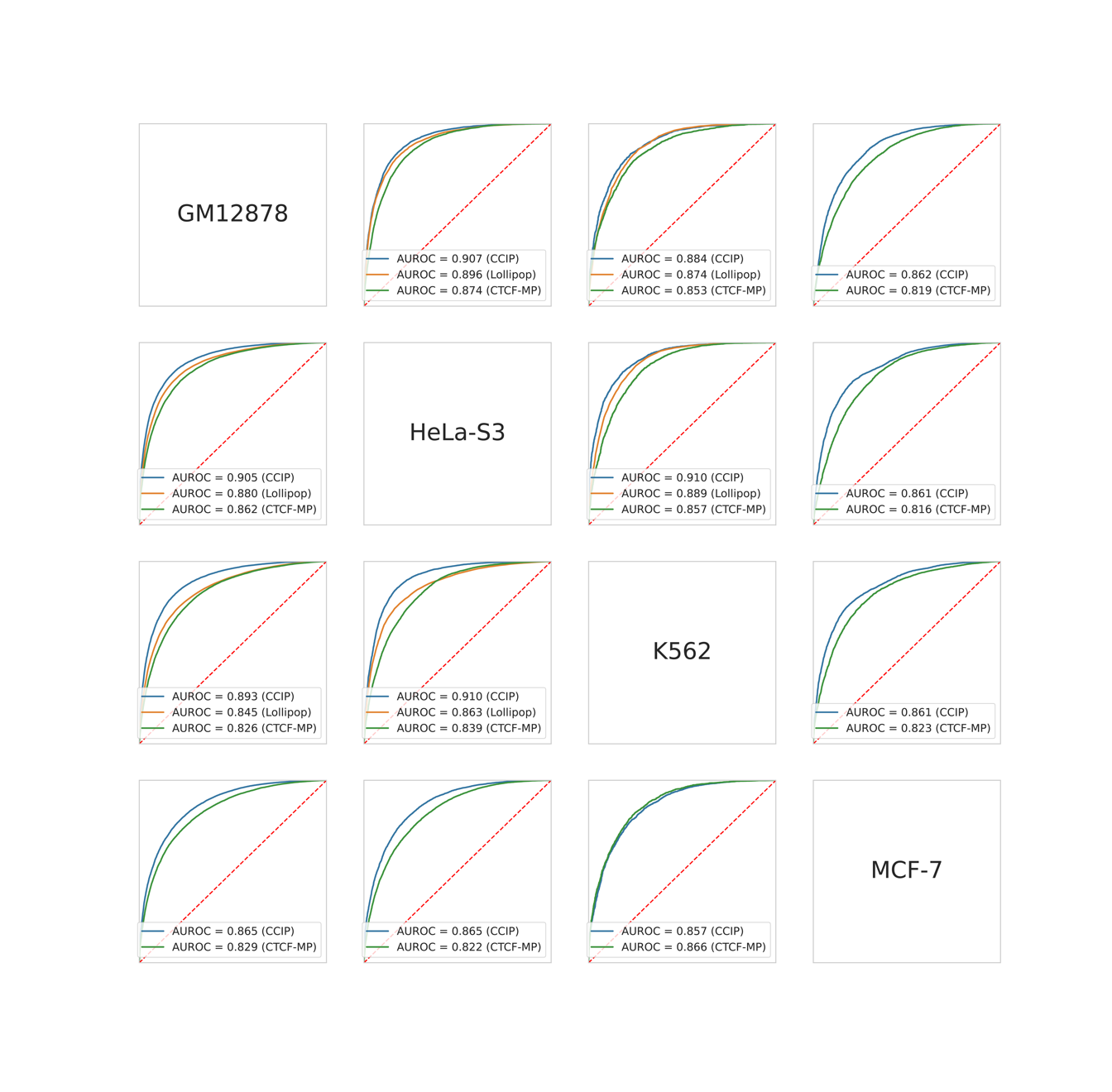


**Fig. S4. ROC curve for across cell type evaluation.** Each row stands for the cell type for model training and each column represents the cell type for model evaluation.


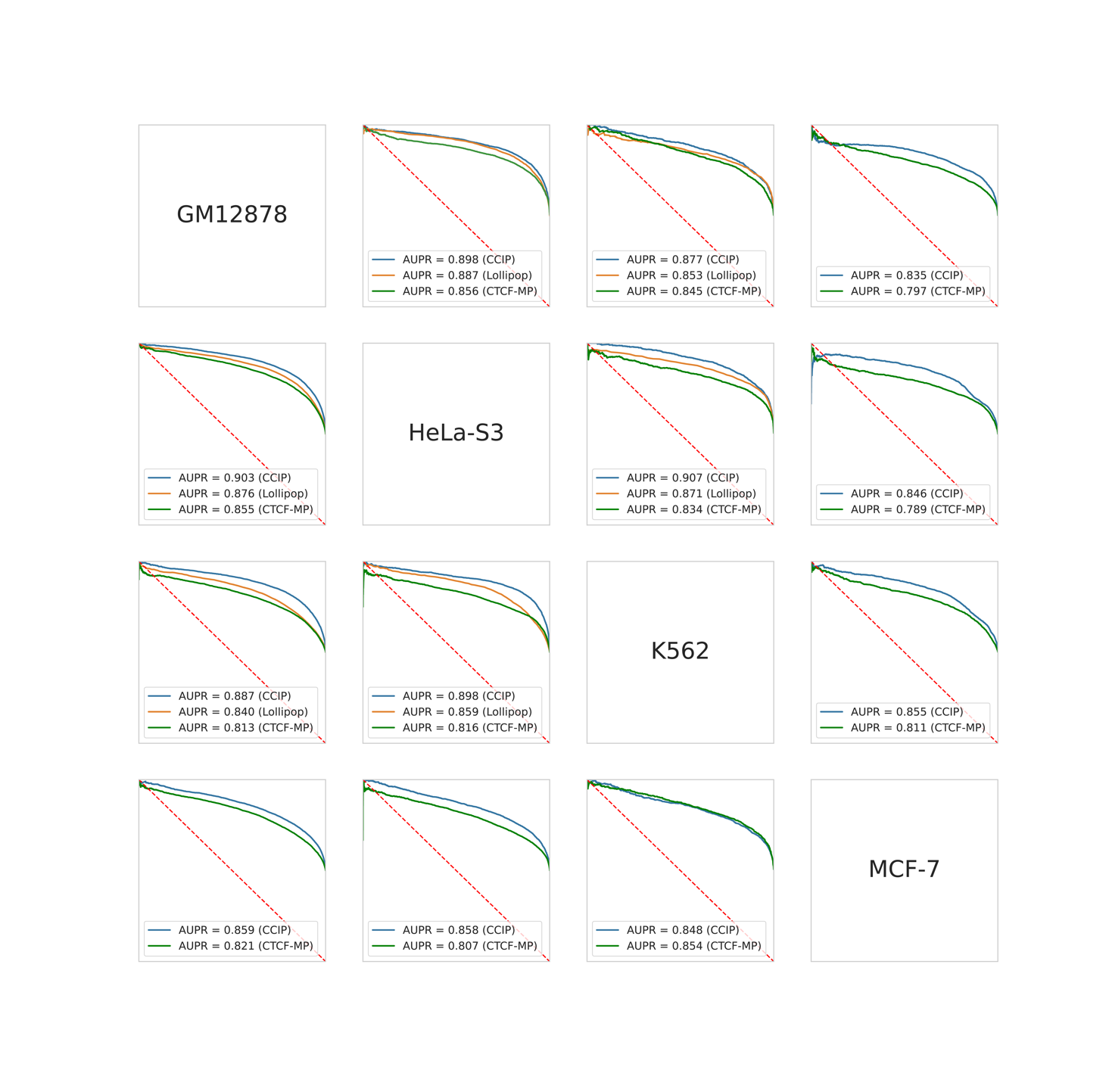


**Fig. S5. PR curve for across cell type evaluation.** Each row stands for the cell type for model training and each column represents the cell type for model evaluation.


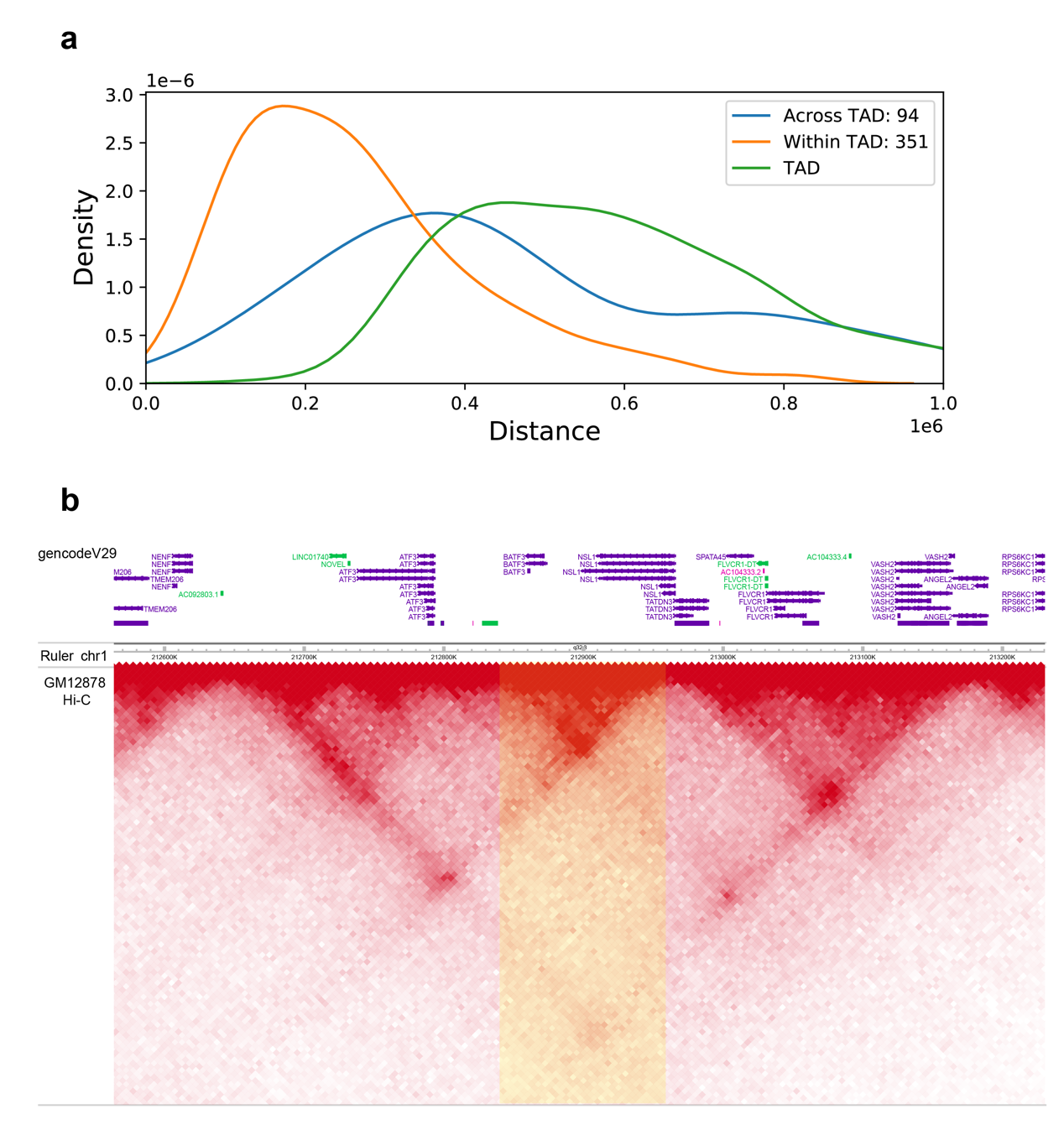


**Fig. S6. CTCF loops serve as the scaffold for enhancer-promoter interaction and transcriptional regulation.** (a) Distribution of distances between the CTCFs and the enhancer-promoter. Within TAD indicates that CTCF, enhancer, and promoter are within one TAD while Across TAD indicates that CTCF, enhancer, and promoter locate different TADs. The green line presents the size distribution of TADs. (b) Hi-C heatmap of GM12878 near BATF3 gene. Yellow vertical bars highlight the genome interval that is discussed in section 2.6 and Fig. 6.


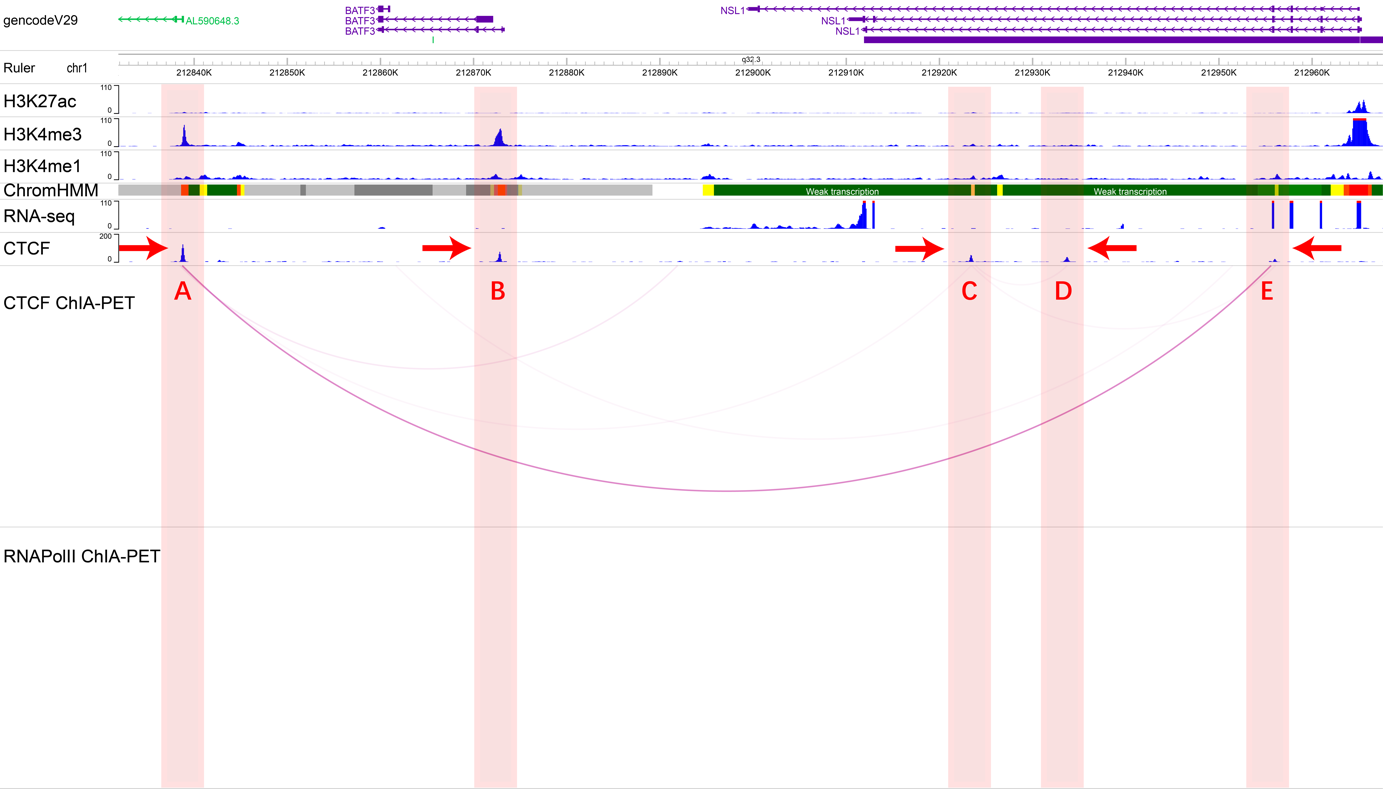


**Fig. S7. Genome browser snapshot of HeLa-S3 near BATF3 gene.**

**
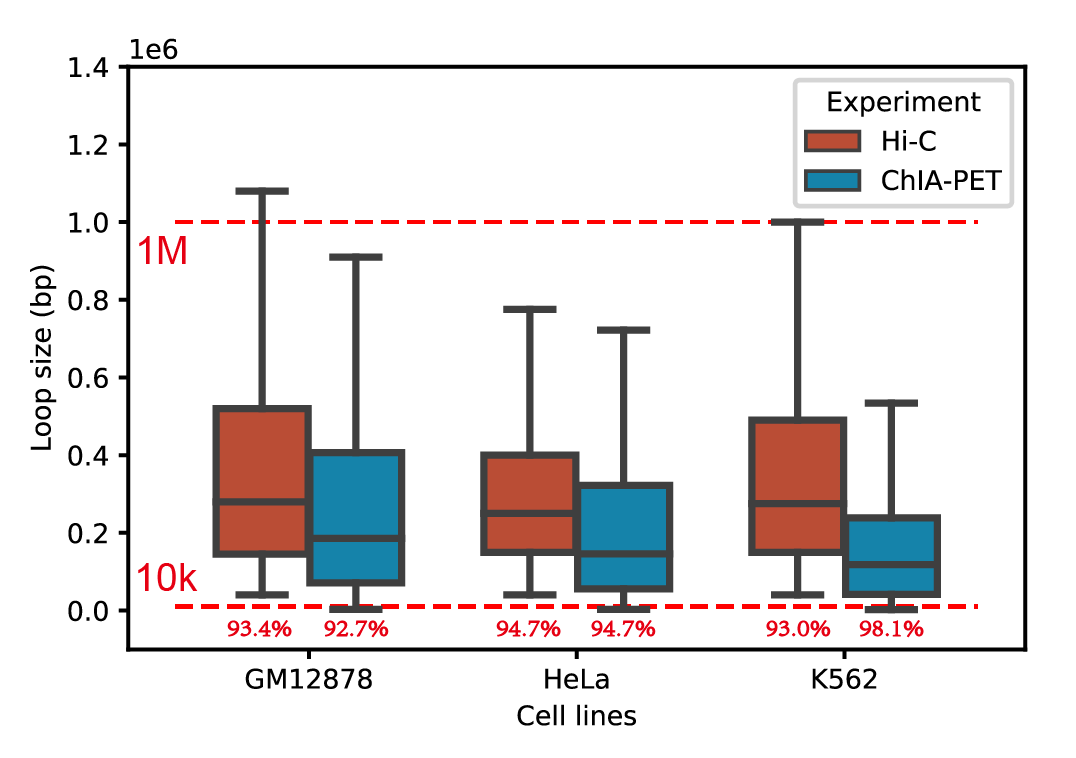
**

**Fig. S8. Size distribution of loops from Hi-C and CTCF ChIA-PET data.** The Red dashed line indicates the loop size interval between 10kb and 1Mb and the red percentage numbers present the proportion of loops in this range. Most of Hi-C loops and ChIA-PET loops are between 10kbp and 1Mbp. Hi-C loops are downloaded from NCBI (accession number: GSE63525).

# Supplementary Tables

**Table S1. Feature combination and its performance (AUPR).**

| **Feature combination** | **Basic model** | **Final model** |
| --- | --- | --- |
| Base | 0.8793 | 0.8803 |
| In-between | 0.6558 | 0.7334 |
| Motif | 0.6998 | 0.7704 |
| Base + In-between | 0.9201 | 0.9237 |
| Base + Motif | 0.8800 | 0.8851 |
| Base + In-between + Motif | 0.9206 | 0.9248 |

**Table S2. Data accession.**

| **Cell type** | **CTCF**  **ChIA-PET** | **CTCF**  **ChIP-seq** | **RAD21**  **ChIP-seq** |
| --- | --- | --- | --- |
| GM12878 | GSE72816 | GSE32465 | GSE29611 |
| MCF-7 | ENCSR000CAD | ENCSR000DML | ENCSR000BTQ |
| K562 | ENCSR000CAC | GSE32465 | GSE32465 |
| HeLa-S3 | GSE72816 | GSE31477 | GSE29611 |
